# Executive Functions and Morphosyntax: Distinguishing DLD From ADHD in French-Speaking Children

**DOI:** 10.3389/fpsyg.2020.551824

**Published:** 2020-10-15

**Authors:** Emily Stanford, Hélène Delage

**Affiliations:** Psycholinguistics Laboratory, Faculty of Psychology and Educational Sciences, University of Geneva, Geneva, Switzerland

**Keywords:** ADHD, DLD, executive functions, omnibus tests, probe tests, differential diagnosis, morphosyntax

## Abstract

Attention deficit hyperactivity disorder (ADHD) is commonly associated with deficits in executive functions executive functions (EF), but children with this disorder frequently demonstrate co-occurring morphosyntactic impairment when assessed using standardized tests. On the other hand, children with developmental language disorder (DLD), a population defined by impaired linguistic functioning, are often diagnosed with comorbid EF deficits. We investigated EF and morphosyntax in 60 French-speaking children aged six to 12: 20 with typical development (TD), 20 with ADHD, and 20 with DLD. To obtain an EF profile for the different cognitive groups, we used standardized tests to assess lower-order EF skills, (i) selective attention and (ii) short-term memory capacity, and higher-order EF skills, (i) working memory capacity and (ii) attention shifting. To test morphosyntax, we used (i) a standardized omnibus test that elicited a variety of complex structures and (ii) a more fine-grained probe test that assessed the production of third person object clitic pronouns, a clinical marker of DLD in French. Children with ADHD and DLD were associated with different EF and morphosyntactic profiles: children in the ADHD group demonstrated higher-order EF weakness and difficulty on the omnibus morphosyntax task, whereas children with DLD showed both lower- and higher-order limitations and struggled with both morphosyntax tasks. Our findings indicate that deficits in morphosyntax are not characteristic of ADHD but that the performance of children with ADHD can mimic morphosyntactic impairment when all-encompassing omnibus tests evaluating various and unpredictable structures are used. If morphosyntax is tested using reliable markers of atypical language development and external cognitive-load factors are optimally reduced, there are significant discrepancies in the observed ADHD-DLD outcomes. Clinical implications that include perspectives for the differential diagnosis of ADHD and DLD are discussed.

## Introduction

Attention deficit hyperactivity disorder (ADHD) is a highly prevalent neurodevelopmental disorder that affects roughly 5–7% of the population. Children with ADHD display atypically high levels of distractibility, hyperactivity and impulsivity (American Psychiatric Association, 2013) and many studies cite executive function (EF) difficulties in this population, i.e., difficulties with selective attention, working memory (WM), inhibition and cognitive control (e.g., Willcutt et al., 2005). Although not a diagnostic marker of the disorder, children with diagnosed attention deficits also commonly present comorbid language difficulties (see meta-analysis by Korrel et al., 2017), which has led some researchers to compare ADHD with another common condition diagnosed in children, developmental language disorder (DLD). Children with DLD are characterized by persistent acquisition difficulties that affect expressive and receptive language, in particular morphosyntax (Leonard, 2014). However, more general cognitive weakness, such as EF limitations, are frequently found alongside language impairment in children with DLD (see meta-analysis by Kapa and Plante, 2015), causing some to question the validity of classifying ADHD and DLD as two distinct disorders.

In what concerns EF abilities in children with ADHD and DLD, there is some evidence that both groups have difficulty executing tasks that require basic attentional processes, such as detecting and selectively attending to relevant stimuli. For example, Richards et al. (1990) found that the presence of ADHD in fourth-, fifth-, and sixth-grade students with learning difficulties led to an increase in the number of errors children made on a visual selective attention task in which they were asked to respond to a central stimulus that is flanked by distracting non-target stimuli. In this study, children with ADHD were more likely to make errors when distractors were adjacent to the target stimulus but not when they were distant, suggesting that the monitoring and inhibiting capacities of children with ADHD are exceeded when asked to focus attention on a small area in the visual field. Similar results were reported by Shalev and Tsal (2003), who also used a flanker task to show that younger children with ADHD (*N* = 5, *M* = 6;5) demonstrate impaired ability to selectively process relevant information by limiting visual attention to a restricted spatial area. However, using a different measure of visual selective attention, a visual cueing task in which participants had to press a button as quickly as possible when a predetermined target was detected, DeShazo Barry et al. (2001) observed that 10-year-old boys with ADHD (*N* = 15) did not behave differently from age-matched TD children. Fewer studies have investigated selective attention capacity in children with DLD, but in Kapa and Plante’s (2015) review of the literature on EF in DLD, they concluded that children with DLD systematically perform below their TD peers on selective attention tasks, findings that were corroborated by Kapa et al.’s (2017) study on preschoolers with DLD (*N* = 26, *M* = 5;9).

Poor performance on WM tasks has also been extensively documented for both children with ADHD and DLD. Meta-analyses from Martinussen et al. (2005) and Kasper et al. (2012) show that children and adolescents with ADHD exhibit deficits on both simple span (storage) and complex span (storage and processing) WM tasks relative to their TD peers. In what specifically concerns complex span, previous work has presented convincing arguments that the core features of ADHD (hyperactivity, inattentiveness and poor inhibition) are related to the ability to perform complex span tasks. For example, Rapport et al. (2009) demonstrated that higher activity levels in 8- to 12-year-old boys (*N* = 12) were related to increases in cognitive load, in particular performing a high-WM demand task in which participants were asked to retain a set of jumbled numbers and a capital letter (e.g., 4 H 6 2) and to then recall the numbers from smallest to largest with the letter at the end of the sequence (2 4 6 H). Using the same WM task, Kofler et al. (2010) showed that attentive behavior decreased as WM demands increased in children with ADHD (*N* = 15, *M* = 9;3), and Alderson et al. (2010) found that greater difficulties performing this particular WM task were related to poorer behavioral inhibitory control in a group of 8- to 12-year-old boys with ADHD (*N* = 14).

There is also substantial evidence for simple and complex span impairments in children with DLD (e.g., Majerus et al., 2009; Delage and Frauenfelder, 2020). In particular, Majerus et al. (2009) found that French-speaking children (*N* = 12, *M* = 8;4) with DLD struggle to perform simple span tasks that required them to reconstruct the serial order of a list of items, findings that were replicated by Delage and Frauenfelder (2020) with a larger sample (*N* = 28, *M* = 8;10). Other studies have shown that children with DLD often perform below their TD peers on complex span tasks, such as the listening span task in which children are asked to retain for subsequent recall the final word of a set of aurally presented sentences while simultaneously making a true-false judgment about each sentence (Weismer et al., 1999; Archibald and Gathercole, 2006). The presence of WM deficits in children with DLD was also confirmed by Kapa and Plante’s (2015) literature review, in which WM deficits were consistently reported for children with DLD in the studies reviewed.

Research on shifting has yielded mixed evidence, with some studies observing slower response times and decreased accuracy on shifting tasks in children with ADHD (Lawrence et al., 2004; Toplak et al., 2008; O’Brien et al., 2010) and others failing to find evidence of impaired shifting (Goldberg et al., 2005; Mulas et al., 2006; Biederman et al., 2007) or attributing the poor performance of children with ADHD on shifting tasks to confounding high-order processes, such as WM and inhibition, that are necessarily involved when completing such tasks (Irwin et al., 2019). However, the variability of these results is likely due to the heterogenous nature of ADHD and the fact that children with this disorder demonstrate a mixed assortment of deficits, which can be problematic when using group averaging to compare the performance of children with ADHD to that of TD children.

Like children with ADHD, attention shifting results are mixed in children with DLD. Im-Bolter et al. (2006) found that children with DLD (*N* = 45, *M* = 10;10) performed comparably to TD peers on a task specifically aimed at measuring one’s ability to mentally alternate between naming and counting numbers, and in Kapa et al.’s (2017) study on preschoolers with DLD, the performance of TD children and children with DLD could not be distinguished on a verbal shifting task that required participants to switch between labeling animals (cat vs. dog) and labeling children (boy vs. girl). Conversely, in the same study, Kapa et al. (2017) reported a significant difference between TD and DLD performance on a non-verbal shifting task, leading the authors to question the reliability of their verbal shifting measure. However, it should be noted that in the other (albeit few) studies investigating attention shifting capacity in children with DLD, non-verbal measures of attention shifting have revealed TD-DLD differences (Farrant et al., 2012; Roello et al., 2015) whereas performance has been similar for verbal shifting measures (Henry et al., 2012).

In what concerns language (and more specifically morphosyntactic) ability, children with DLD are known to demonstrate difficulties with non-canonical structures that do not follow the order subject-verb-object in languages such as French or English. For example, there is a multitude of crosslinguistic evidence demonstrating that children with DLD struggle to comprehend and produce object relative clauses, whereas the comprehension and production of subject relative clauses is markedly better (e.g., see Delage et al., 2008 for French, Novogrodsky and Friedmann, 2006 for Hebrew, Contemori and Garraffa, 2010 for Italian, Jensen De López et al., 2014 for Danish, Stavrakaki, 2001 for Greek, Frizelle and Fletcher, 2014 for English). This asymmetry extends to object and subject questions, and in languages such as French, children with DLD tend to circumvent object movement by producing *in situ* questions (1) in which the object remains in its postverbal position (a fully grammatical option in French) rather than more complex *ex situ* questions (2) in which the object is fronted and the canonical word order is disrupted (Cronel-Ohayon, 2004; Hamann, 2006; Jakubowicz, 2011).


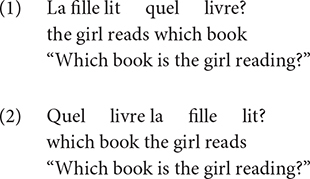


Also in French-speaking children with DLD, the delayed acquisition of third-person (3p) object clitic pronouns (3), which appear preverbally in Romance languages, is characteristic of the disorder and considered a clinical marker of atypical language development, as low production rates of such clitics reliably distinguish French-speaking individuals with DLD from those with typical development. This is in contrast to less morphosyntactically complex pronouns, such as subject and reflexive pronouns and first- and second-person object pronouns, which develop with relatively little difficulty (Paradis et al., 2003; Chillier-Zesiger et al., 2006; Tuller et al., 2011; Delage et al., 2016). Common non-target behavior reported for children with DLD in elicitation studies includes ungrammatical omissions of the 3p object clitics, productions with a gender error on the object clitic, or productions in which a lexical noun phrase is used instead of a more pragmatically appropriate object clitic (Tuller et al., 2011; Stanford et al., 2019).


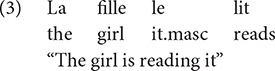


While morphosyntax in ADHD has not been investigated to the same extent as in DLD, several studies nonetheless indicate that children with ADHD present morphosyntactic weakness. For example, Love and Thompson (1988) found that in a large sample of English-speaking preschool children (*N* = 116, *M* = 5;0) demonstrating attention difficulties, many also displayed previously undetected language impairment when tested using the first edition of the Reynell Developmental Language Scales (Reynell and Curwen, 1977), a standardized battery created to identify language delay and impairment in young children. Similar results were reported by Cohen et al. (1993) and Cohen et al. (2000) when they tested older children (4–12 years old in the 1993 study and 7–14 years old in the 2000 study) who met the diagnostic criteria for ADHD on receptive and expressive morphosyntax using a variety of standardized tests taken from different batteries, as well as by Kim and Kaiser (2000), who used the Test of Language Development-2 Primary (Newcomer and Hammill, 1988) to assess receptive and expressive morphosyntax in 6–8 years old (*N* = 11) diagnosed with ADHD. More recently, Korrel et al.’s (2017) meta-analysis of 21 studies assessing language via standardized tests in children with ADHD confirmed that expressive language difficulties are widely attested for this population, although findings for morphosyntax were mixed.

It is also worth noting that several crosslinguistic studies have examined the morphosyntactic competences of school-aged children with ADHD using Bishop’s (2003) Children’s Communication Checklist-Second Edition (see Fortea et al., 2018 for Spanish, Geurts and Embrechts, 2008 for Dutch, Helland et al., 2012 for Norwegian, and Väisänen et al., 2014 for Finnish). Again, findings are inconsistent as Väisänen et al. (2014) and Fortea et al. (2018) report morphosyntactic weakness for children with ADHD when compared to their TD peers, whereas children with ADHD could not be distinguished from the TD group in the studies by Geurts and Embrechts (2008) and Helland et al. (2012). Furthermore, Helland et al. (2012) compared the scores of children with ADHD to those of age-matched children with DLD (*N*_ADHD_ = 21, *M*_ADHD_ = 10;1 and *N*_DLD_ = 19, *M*_DLD_ = 8;7) and found that while there was an overlap between the two groups for some language subscales included in the questionnaire (e.g., semantics, coherence, use of context), the two groups could be statistically discriminated based on the morphosyntactic scores that emerged: morphosyntax was ranked as intact by parents of children with ADHD but as impaired by parents of children with DLD. Helland et al. (2012) argued that this result could not be due to the age difference between the two clinical groups as no correlation was found between age and language scores in the study. It should also be pointed out that although children with ADHD and TD children could not be distinguished based on parental rankings of morphosyntax in Helland et al. (2012), children with ADHD did nonetheless demonstrate poorer performance on the morphosyntactic subscales than their TD peers, a pattern that also emerged in Geurts and Embrechts (2008). It is thus possible that children with ADHD demonstrate modest morphosyntactic difficulties, which would explain the results reported by Väisänen et al. (2014) and Fortea et al. (2018), but that these difficulties are not comparable in severity to those displayed by children with DLD.

While there seems to be a strong argument in favor of morphosyntactic weakness in children with ADHD, it should be pointed out that morphosyntax was always assessed using standardized evaluations in the studies presented above. Norm-referenced tests provide important information about a child’s language progress in relation to his or her peers and have high validity and reliability in most cases, but they also have some shortcomings. One disadvantage of such omnibus tests is that due to the heterogeneous nature of the structures tested, they can be quite uninformative when it comes to pinpointing strengths and weaknesses on specific morphosyntactic structures. For example, in Cohen et al. (2000) study, the Word Structure subtest from the Clinical Evaluation of Language Fundamentals-Revised (CELF-R; Semel et al., 1995) was one of the measures used to assess expressive morphosyntax, and within this subtest, up to 17 morphosyntactic rules are included in the 32 items. It is unclear how symptoms of ADHD, such as high levels of distractibility, interact with performance on such evaluations.

Using more fine-grained measures, Redmond (2005) found that while both English-speaking DLD and ADHD children (*N* = 10, *M* = 6;7 for DLD and *N* = 10, *M* = 6;11 for ADHD) performed poorly on tasks measuring sentence recall when compared to age-matched TD children, children with DLD demonstrated greater difficulty than the other two groups on this task, resulting in a TD > ADHD > DLD pattern of performance. Sentence recall difficulties in ADHD have been reported in other studies (Love and Thompson, 1988; Cohen et al., 1989, 1993, 1998, 2000; Tannock and Schachar, 1996; Barkley, 1997; Purvis and Tannock, 1997; Tirosh and Cohen, 1998; Kim and Kaiser, 2000), but Redmond (2005) suggests that this may in fact be influenced by external factors, such as distractibility, because such tasks represent a “rote, decontextualized, non-meaningful” activity (p. 122), whereas the difficulties of children with DLD could be attributed to true morphosyntactic impairment. In the same study, on a task that measured the elicited production of past tense morphology, a clinical marker of DLD in English, Redmond reported a much clearer asymmetry between ADHD and DLD performance: children with ADHD performed comparably to TD children while difficulty with the task was characteristic of children with DLD (TD = ADHD > DLD). In a later study in which Redmond et al. (2011) assessed 7-to-8 year old English-speaking children with ADHD and with DLD on four clinical markers of morphosyntactic impairment, the authors reported that children with ADHD performed comparably to their TD peers and could be differentiated from the DLD group based on performance on these tasks. Thus, there is some evidence that when the appropriate psycholinguistic indices of DLD are used to assess morphosyntax, children with ADHD and DLD can be distinguished: children with ADHD no longer display morphosyntactic difficulties when assessed using more fine-grained measures, whereas morphosyntactic weakness remains regardless of task type for children with DLD.

To our knowledge, despite the seeming overlap in the EF and morphosyntactic profiles of children with ADHD and DLD, no single study has compared these two groups on these measures. Furthermore, we are unaware of any study that has investigated the validity of using standardized morphosyntax evaluations with children with ADHD. Redmond (2005, 2016) and Redmond et al. (2011) encourage the use of probe tests that measure clinical markers of DLD when assessing the morphosyntactic capacities of children with ADHD, but no comparison with standardized tests has been made in previous studies. Finally, studies on EF and morphosyntax in French-speaking children with ADHD and DLD are totally absent from the literature.

In order to better understand how the TD-ADHD-DLD cognitive and morphosyntactic profiles are similar and how they diverge, we compare the performance of these three groups on three EF tasks, (i) selective attention, (ii) working memory and (iii) attention shifting, and on two morphosyntax tests, (i) an omnibus test that evaluates the production of a variety of complex morphosyntactic structures and (ii) a more fine-grained probe test that specifically assesses the production of 3p object clitics, a clinical marker of DLD in French. Based on the widely documented EF deficits for both children with ADHD and DLD, we predict that both clinical groups will experience comparable difficulties with the EF tasks and that the performance of these two groups will thus be lower than that of our TD control group for our EF measures (TD > ADHD = DLD). As for performance on the morphosyntax task, children with DLD should demonstrate significant difficulty when compared to their TD peers as severe and persistent morphosyntactic impairment is characteristic of the disorder. Reports of morphosyntactic performance are variable for children with ADHD. For example, there is some evidence that when children with ADHD are tested on clinical markers of DLD, they perform like their TD peers (Redmond, 2005; Redmond et al., 2011). However, there are also consistent findings that children with ADHD perform poorly on standardized omnibus morphosyntax assessments. We expect this to hold true in our study, although we do not predict difficulties to be as severe as in the case of the DLD group (TD > ADHD > DLD).

Next, focusing specifically on our two morphosyntax measures, we ask whether different types of morphosyntax tests, standardized omnibus tests vs. probe tests, can be used by clinical practitioners to reliably identify morphosyntactic impairment in children with ADHD as it is uncertain if the poor performance of this population on morphosyntax assessments is due to actual morphosyntactic weakness, or to external factors related to their EF deficits (e.g., test duration or unpredictability of task). Our prediction is if children with ADHD have difficulties in morphosyntax similar to those found in DLD, they should be at a disadvantage for both tasks. Conversely, if poor performance in children with ADHD is limited to the standardized omnibus task that varies unpredictable structures, this would corroborate Redmond’s findings for English-speaking children and would give an indication of the discriminant validity of more fine-grained morphosyntactic measures that specifically assess performance on clinical markers of DLD.

To summarize, we ask the following two questions:

(i) How does the performance of TD children, children with ADHD and children with DLD compare on our different EF and morphosyntax tasks?
(ii) Do children with ADHD perform similarly on both morphosyntax tasks?

## Methodology

### Participants

Sixty French-speaking children aged 6–12 participated in this study: 20 children with DLD (*M* = 8;6, SD = 1;7), 20 children with ADHD (*M* = 8;10, *SD* = 1;5), and 20 TD children (*M* = 8;6, *SD* = 1;4). Participants in the DLD group had been officially diagnosed by a qualified speech-language therapist (SLT), and, as we were specifically interested in investigating if children with ADHD demonstrate morphosyntactic weakness similar to that of children with DLD, only children with DLD with documented deficits in morphosyntax were included in this study. We verified these deficits via correspondence with the SLTs directly involved in the children’s intervention services, and two children diagnosed with DLD were excluded from participation in the study because they did not have deficits in morphosyntax.

Five children in the DLD group had been diagnosed with comorbid attention difficulties. While an anonymous reviewer suggested that children with comorbid DLD and ADHD should have been excluded from the study, we felt that to do so would not reflect the reality of the situation and that applying overly strict participation criteria would result in artificial clinical groups. However, participants with comorbid deficits were examined in case-by-case fashion and in consultation with practitioners and parents in order to establish if a primary deficit was present, which ultimately determined group placement. In three of these children, attention deficits were deemed very mild by the SLTs involved in their intervention services, which was also confirmed by parental responses on the Conners Comprehensive Behavior Rating Scales for parents (CBRS, Conners, 2008), a tool designed to assist clinicians in evaluating children for ADHD. In the other two participants, attention difficulties were more clearly present, but impairment in morphosyntax was considered the primary deficit by the relevant SLTs and these children were thus permitted to participate in the study as part of the DLD group. All children in this group were receiving SLT services on a regular basis (one to two sessions per week). Children in the ADHD group had also been formally assessed and diagnosed by a psychologist. Additionally, parents of the participants were asked to complete the CBRS, allowing us to classify our participants with ADHD into one of two groups: (i) predominately hyperactive/impulsive (*n* = 2) and (ii) mixed hyperactive and inattentive (*n* = 18). Two of the participants in the ADHD group had suspected but undiagnosed language difficulties, for which they were not receiving therapy. As the testing sessions were optimally scheduled to take place over weekends or during school holidays, only four of the 20 children with ADHD had taken prescribed medication at the time of testing. As the EF scores (described below) of these four children did not significantly impact the ADHD group average (i.e., the ADHD average on the EF measures did not differ when these children were removed), we did not exclude these participants from the study. The TD children who participated in this study were all attending conventional schools and had no reported history of language impairment or attention difficulties and had never received speech-language therapy or behavioral treatment.

Children with DLD were recruited by contacting SLTs in French-speaking Switzerland and children with ADHD were recruited through parent associations in the same area. Because of the multilingual nature of Switzerland, bilingual children were included in this study provided acquisition of French occurred before the age of three. In total, 13 bilingual children took part in this study (9 in the DLD group, 1 in the ADHD group, 3 in the TD group). As half of the participants with DLD were bilingual, we performed an independent *t*-test on their standardized morphosyntax scores (test described below) to verify that there was no significant difference on morphosyntactic performance between monolingual and bilingual participants in this group, *p* = 0.48. This was not done for the ADHD and TD participants due to the small number of bilingual children in those groups. An anonymous reviewer highlighted that children with DLD and bilinguals have shown significant overlap in morphosyntactic errors in previous studies and was concerned that similar performance of mono and bilinguals in the DLD group on the standardized morphosyntax measure may not be a meaningful way to disentangle bilingualism effects from language impairment. For this reason, independent *t*-tests were also performed on the DLD probe test (described below) scores, as impaired mastery of 3p object clitic pronouns has been shown to be a reliable detector of language impairment in French-speaking children (Tuller et al., 2011). Furthermore, there is some evidence that bilinguals with and without DLD can be distinguished based on their use of 3p object clitics (Jacobson, 2012). The results of our supplementary analyses confirmed that there were *no* significant differences between the mono and bilingual participants in the DLD group for any of the relevant measures (*p* = 0.45 for the production of target 3p object clitics, *p* = 0.33 for the use of a lexical DP instead of the target clitic, *p* = 0.81 for the omission of the target clitic and *p* = 0.13 for the production of 3p object clitic with a gender error). We believe that the similar performance of the two groups on both the standardized and the probe test provides sufficient evidence that the bilinguals in the DLD group represent true cases of language impairment.

Finally, all children were assessed for non-verbal reasoning using Raven’s Progressive Matrices (Raven et al., 1998). While all cognitive groups were within the normal range (≥10th percentile), independent *t-*tests revealed that both the TD and ADHD groups were significantly better than the DLD group for this measure, *t*(38) = 4.59, *p* < 0.001, *d* = 1.21 for TD vs. DLD *t*(38) = 2.86, *p* = 0.01, *d* = 0.84 for ADHD vs. DLD. We considered this unproblematic as findings from prior research suggest that children with DLD typically perform below their TD peers on non-verbal tasks (see Leonard, 2014 for an overview), and the definition of DLD as described by Bishop et al. (2017) specifies that non-verbal delays are no longer exclusionary for a DLD diagnosis, provided the delays are not related to diagnosed intellectual disability. One child with DLD (age = 11;3) had a particularly low non-verbal reasoning score (<5th percentile) despite having not been diagnosed with comorbid difficulties and successfully attending a mainstream school. To ensure that DLD group performance was not being affected by this participant, all analyses presented in section “Results” were rerun removing this child’s scores. As excluding this participant yielded no significant effect changes, we allowed him to remain in the sample. Thus, the results presented in 3 were obtained via analyses that included this participant. Table 1 displays descriptive summary data for each group of participants.

**TABLE 1:** Summary of participant information according to cognitive group.

Cognitive group	*N*	Bilingual: *N*	Gender	Age range (year; month)	Age: *M* (*SD*)	NVR: *M* (*SD*)
DLD	20	9	8F, 12M	6;5 – 11;3	8;6 (1;7)	−0.67 (0.98)
ADHD	20	1	7F, 13M	6;3 – 10;7	8;10 (1;5)	0.21 (0.97)
TD	20	3	12F, 8M	6;10 – 11;7	8;6 (1;4)	0.51 (0.61)

*NVR, non-verbal reasoning.*

Approval for this study was obtained from both the Ethics Committee of the Faculty of Psychology at the University of Geneva and the Cantonal Ethics Committee for Research in the canton of Geneva, Switzerland. Parents of all participants gave informed, written consent for their child’s participation in this study.

### Materials

#### EF Tests

##### Selective attention

We evaluated visual selective attention capacity in our participants via the Sky Search task (TEA-ch, Manly et al., 2006), which requires subjects to identify and circle pairs of identical “spacecrafts” from a page of visually similar stimuli while ignoring all distracting items. Of the 49 displayed spacecrafts, 20 corresponded to sets of identical pairs. Speed and accuracy were the measures of interest, and an age-corrected normative score adjusted for motor control was calculated for each participant.

##### Working memory

Working memory was assessed using a classic digit recall task (WISC-IV, Wechsler, 2003) in which participants are asked to recall a string of digits, either in the same or reverse order as read aloud by the examiner. String length grows successively, and testing ends when the participant fails to correctly repeat two consecutive strings of the same length. Each child received a forward and backward digit span score, with forward digit span indicating one’s ability to temporarily store verbal information (i.e., simple span capacity) and backward digit span indicating one’s ability to temporarily store verbal information while performing an additional processing task (i.e., complex span capacity). For both simple and complex span, a total score was calculated that combined the longest correctly repeated sequence of digits (max = 9) and the number of correctly repeated test items (max = 16). Therefore, a child who was able to correctly repeat a sequence of up to four digits and who had correctly repeated digit sequences for six items would receive a total score of 10.

##### Attention shifting

**FIGURE 1:**
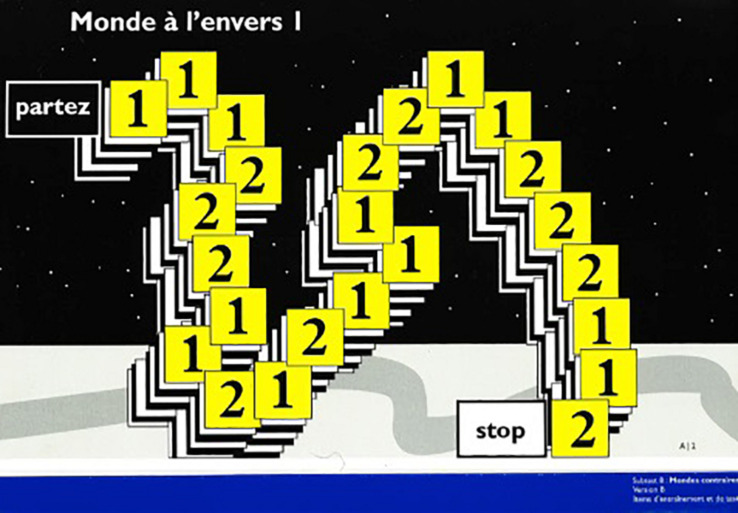
An example of an Opposite World condition (*Monde à l’envers* in French) in which participants had to say the opposite of what they saw (“two” when they saw the digit 1 and “one” when they saw the digit 2).

Performance on the Opposite Worlds task (TEA-ch, Manly et al., 2006) served as an indicator of attentional control/shifting. This task involves two conditions. In the Same World condition, participants follow a path containing the digits 1 and 2, naming each digit out loud (i.e., saying “one” when they see the digit 1 and “two” when they see the digit 2. Conversely, in the Opposite World condition (Figure 1), the participants were informed that they must say “one” when presented with the digit 2 and “two” when presented with the digit 1. Errors resulted in a time penalty as participants could not proceed to the subsequent digit until the error had been corrected. In total, the participants saw two worlds with Same World rules and two worlds with Opposite World rules that were presented in the following order: Same World 1 – Opposite World 1 – Opposite World 2 – Same World 2. We were particularly interested in two time measures, the average time it took the participants to complete the two Same World conditions, which indicated processing speed capacity, and the average time it took participants to complete the two Opposite Worlds conditions, which revealed one’s ability to inhibit a prepotent response and adopt a new set of rules.

#### Morphosyntax Tests

##### Standardized omnibus test

**FIGURE 2:**
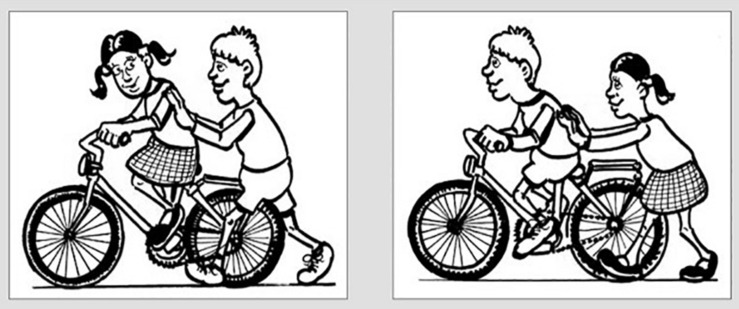
Illustration corresponding to the example item in (4).


To evaluate the general morphosyntactic capacities of our participants, each child was tested using the BILO 3C, a subtest of the *Bilan Informatisé de Langage Oral* (Khomsi et al., 2007), which is a computerized oral language assessment designed to measure expressive morphosyntax in school-aged French-speaking children and adolescents. In this test, the child is shown an image that is briefly described by an audio recording, immediately followed by a second image and a recorded sentence stem. Using the first description as a model, the child is then incited to describe the second image by completing the sentence using the appropriate structure (4). The test contains 29 items that necessitate the production of a variety of complex morphosyntactic forms, such as passives (*n* = 2), future tense (*n* = 2), past tense (*n* = 3), plural verb conjugations (*n* = 4), irregular plural nouns (*n* = 4), contracted articles (*n* = 3) etc. Correct responses are given 1-2 points with a maximum score of 36 points, and raw scores are converted into standard scores. An example item is shown in Figure 2.

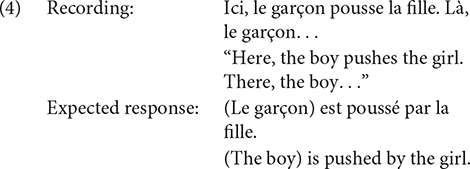


##### Probe test

**FIGURE 3:**
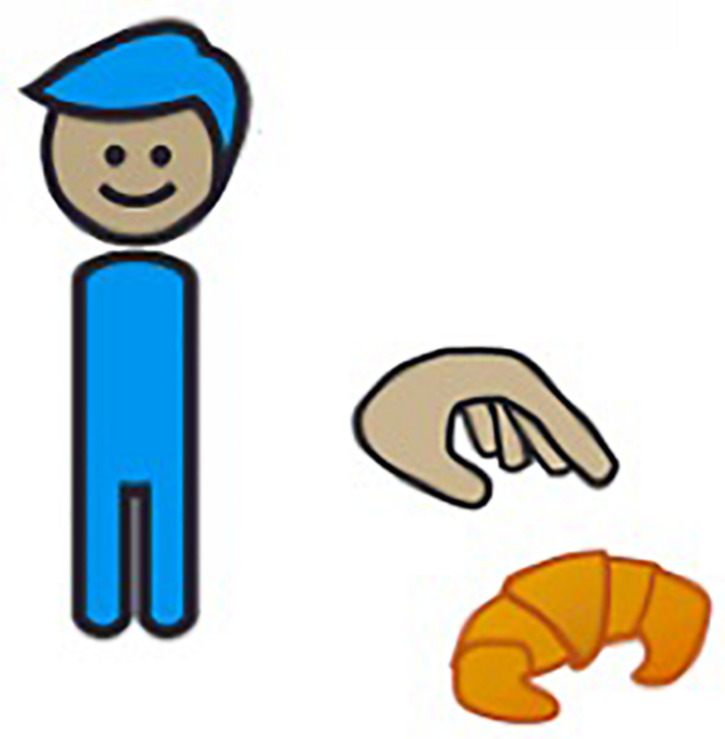
Illustration corresponding to the example item in (5).

Next, we zoomed in on the production of 3p object clitics pronouns, as the delayed acquisition of these structures is considered a clinical marker of DLD in French-speaking individuals, and low production rates of such clitics reliably distinguish individuals with DLD from those without the disorder in French (Paradis et al., 2003; Tuller et al., 2011). To test the production of 3p object clitics, we used a Production Probe for Pronoun Clitics task (adapted from Tuller et al., 2011; Delage et al., 2016) that required participants to respond to a question about an image that appeared on a computer screen. The task, which elicited 3p nominative and accusative clitic pronouns, contained three training items, twelve test items and four distractors (see Table 2). Both animate and inanimate arguments were used as referents for the 3p object clitics, and items contained accusative and nominative pronouns. A sample item is provided in (5) along with the corresponding image (Figure 3).


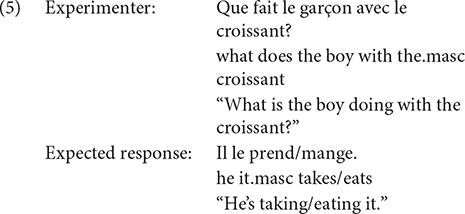


**TABLE 2:** Summary of the clitic pronouns elicited by the probe task.

	Nominative	Accusative
**3p**		
Masculine	il (6)	le (5)
Feminine	elle (6)	la (5) l’ (2)^1^

*^1^This is the French gender-neutral elided clitic that is used before a verb that begins with a vowel or a mute *h*.*

###### Coding criteria

The productions for the probe test were coded under five main categories: target 3p object clitic (the same as the expected response in Example 5), non-target object clitic, such as ones containing a gender error (6), a lexical DP instead of a 3p object clitic (7), an omitted object clitic (8), and other responses (9). Responses 6-9 are based on the example item provided in 5 and Figure 3. The responses in examples 6 and 8 are ungrammatical, indicated by an asterisk, while response 7 is grammatical but infelicitous in the context, indicated by the preceding question mark, as it unnecessarily repeats the full DP. It should be noted that the response in (6) is incorrect because it contains a gender error within the context of the sample item in (5), not because it is structurally ungrammatical.


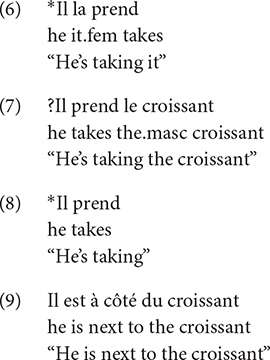


### Procedure

In a one-to-one setting, participants completed three EF tests and two expressive morphosyntax tests that lasted approximately 45 min. The assessment was administered by the main author and graduate students in an SLT postgraduate program in Geneva, Switzerland and took place either at the home of the child or in a consulting room at a private SLT practice.

## Results

### Preliminary Analyses

Before examining how the different groups performed on the EF and morphosyntax tasks, we wanted to first investigate if performance on these tasks was related to non-verbal reasoning as non-verbal reasoning was weaker in the DLD group than in the other two groups. Correlation analyses revealed that non-verbal reasoning was not related to performance on any of the EF or morphosyntax tasks in the DLD group. It was, however, related to EF performance in the ADHD group for three of the six measures. As for morphosyntax, there was also a significant positive relationship between ADHD performance on the omnibus test and non-verbal reasoning skills. These results are summarized in Table 3.

**TABLE 3:** Correlation analyses for non-verbal reasoning and the relevant EF and morphosyntax measures.

	Non-verbal reasoning		
	
	TD	ADHD	DLD
**Selective attention**			
Accuracy	0.10	0.051*	–0.11
Speed	–0.30	−0.047*	–0.12
**Working memory**			
Simple span	–0.12	0.56**	–0.04
Complex span	0.24	0.12	–0.07
**Attention shifting**			
Same World speed	–0.04	–0.08	–0.13
Opposite World speed	–0.22	–0.26	–0.02
**Omnibus test**			
*Z*-score	0.09	0.45*	0.05
**Probe test**			
Total correct clitics	–0.22	0.10	0.06

*p ≤ 0.05. **p ≤ 0.01.

### Question 1: How Did the Different Groups Perform on the EF and Morphosyntax Tasks?

The first thing we compared was how the different cognitive groups performed on our three EF tasks. Based on the fact that children with ADHD and DLD have documented EF limitations, we predicted that the performance of both clinical groups would be significantly lower than that of age-matched TD children. One-way between-groups ANOVAs were run with cognitive group (TD, ADHD, DLD) as the between subjects factor, and a significant effect of cognitive group was found for five of the six EF measures: *F*(2,57) = 5.81, *p* = 0.01, η^2^ = 0.16 for speed on the selective attention task; *F*(2,57) = 6.48, *p* = 0.003, η^2^ = 0.19 for Same World speed; *F*(2,57) = 11.15, *p* < 0.001, η^2^ = 0.28 for Opposite World speed; *F*(2,57) = 9.11, *p* < 0.001, η^2^ = 0.24 for simple span; *F*(2,57) = 4.69, *p* = 0.01, η^2^ = 0.14 for complex span. *Post hoc* Tukey HSD tests revealed that TD children were significantly better than children with DLD for all five of these measures. For selective attention, we also considered an additional measure that adjusted performance for motor speed (score calculated based on the number of identified target pairs minus time taken to complete a basic motor speed task) and found that the significant TD-DLD difference remained (*p* = 0.002, *d* = 0.36). TD children were significantly better than children with ADHD for Opposite World speed, and there was a strong tendency for TD children to be better than children with ADHD for Same World speed. Children with ADHD and DLD could only be statistically distinguished for simple span, with children with ADHD having a significantly higher simple span score than children with DLD. As for the measure of accuracy in the selective attention task, all groups performed close to ceiling and could not be statistically distinguished. These results are summarized in Table 4.

**TABLE 4:** Summary of EF scores for the three cognitive groups.

	Selective attention				Attention shifting				Working memory			
			
	Number of correct		Response time (s)		Average time (s) for		Average time (s) for		Simple span total		Complex span total	
						
	targets/20		per target		Same World		Opposite World		score/25		score/25	
						
	*M% (SD)*	Comparison	*M* time *(SD)*	Comparison	*M* time *(SD)*	Comparison	*M* time *(SD)*	Comparison	*M* score *(SD)*	Comparison	*M* score *(SD)*	Comparison
TD	18.10 (2.40)	**ns**	5.76 (1.38)	**	27.64 (6.86)	**	33.12 (8.23)	***	12.30 (3.03)	***	10.00 (2.51)	**
DLD	16.45 (2.87)		10.16 (5.20)		38.01 (11.87)		49.70 (15.85)		8.60 (2.09)		7.75 (2.07)	
TD	18.10 (2.40)	**ns**	5.76 (1.38)	**ns**	27.64 (6.86)	ns/* (*p* = 0.053)	33.12 (8.23)	***	12.30 (3.03)	**ns**	10.00 (2.51)	**ns**
ADHD	17.95 (2.74)		8.02 (4.66)		32.84 (7.60)		44.01 (12.16)		11.70 (3.53)		8.25 (2.69)	
ADHD	17.95 (2.74)	**ns**	8.02 (4.66)	**ns**	32.84 (7.60)	**ns**	44.01 (12.16)	**ns**	11.70 (3.53)	**	8.25 (2.69)	**ns**
DLD	16.45 (2.87)		10.16 (5.20)		38.01 (11.87)		49.70 (15.85)		8.60 (2.09)		7.75 (2.07)	

*p ≤ 0.05. **p ≤ 0.01. ***p ≤ 0.001.

**FIGURE 4:**
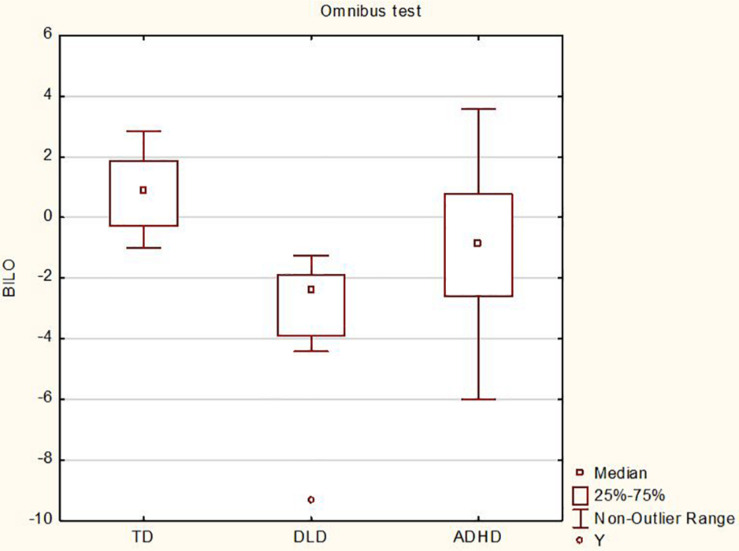
Results for the omnibus test for the three cognitive groups.

Next, we examined how the different cognitive groups performed on the standardized omnibus task. As DLD is a disorder characterized by morphosyntactic impairment, we expected children in this group to perform poorly when compared to TD children. For children with ADHD, one consistent finding is that they tend to perform poorly on standardized omnibus tests of morphosyntax, which is what we also expected to observe. From a qualitative point of view, TD performance was the best of the three groups with all TD children performing within the normal range (no more than 1 SD below the mean), DLD performance was the poorest of the three groups with all children performing within the impaired range (at least 1.5 SD below the mean), and ADHD performance fell between the other two groups displaying a large degree of variability, with nine children performing at least 1.5 SD below the mean and 11 performing no more than 1 SD below the mean. Using a one-way between-groups ANOVA to compare the different *z*-scores (*M*_*TD*_ = 0.86, *M*_DLD_ = −2.96, *M*_ADHD_ = −0.94), we observed a significant main effect of cognitive group, *F*(2,57) = 21.34, *p* < 0.0001, η^2^ = 0.43 (see Figure 4). *Post hoc* Tukey tests confirmed that TD children performed better than both children with DLD and children with ADHD on this test, *p* = 0.0001, *d* = 2.71 for TD vs. DLD and *p* = 0.01, *d* = 0.81 for TD vs. ADHD. There was also a significant difference between ADHD and DLD performance (ADHD > DLD), *p* = 0.003, *d* = 1.72.

**FIGURE 5:**
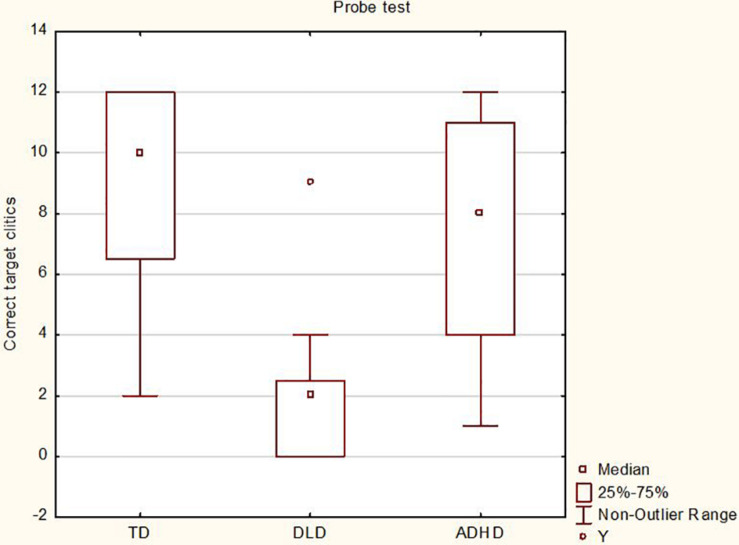
Results for the probe test for the three cognitive groups.

As for the probe task, all three cognitive groups were at ceiling for the production of subject pronouns (>90% accuracy). Regarding the production of 3p object clitic pronouns, correct target clitics were produced 76% of the time by TD children and 61% of the time by children with ADHD, even if scores were quite widely spread in these two groups. This wide distribution may reflect the fact that the number of correctly produced target clitics was significantly related to age in these two groups, *r*(17) = 0.54, *p* = 0.01 for TD and *r*(17) = 0.57, *p* = 0.01 for ADHD, with younger children in both groups producing fewer 3p clitics. Children with DLD produced correct target clitics 15% of the time, and there was no significant correlation between clitics score and age, *r*(17) = 0.01, *p* = 0.95. The reviewer suggested that the production of 3p object clitics may have also been related to non-verbal reasoning but correlation analyses did not reveal a significant link in any of the groups, *r*(17) = 0.09, *p* = 0.36 for TD, *r*(17) = 0.10, *p* = 0.67 for ADHD and *r*(17) = 0.06, *p* = 0.80 for DLD. Mixed models for logistic regression were run using R (R Core Team, 2020) with cognitive groups as fixed factors and items and subjects as random factors as backward elimination procedure indicated that this was the most parsimonious model. As shown in Table 5, the analyses revealed a significant main effect of cognitive group for the production of correct target clitics, χ^2^(2, *N* = 60) = 44.29, *p* ≤ 0.001 (see Figure 5), with *post hoc* Tukey tests showing that TD children and children with ADHD could not be distinguished from one another but that both groups produced significantly more target object clitics than children with DLD.

**TABLE 5:** Summary of *post hoc* Tukey test results for object clitic production following the mixed models for logistic regression analyses.

Contrast	Estimate	*SE*	*z*-ratio	*p*-value
**Object clitics**				
ADHD-DLD	3.08	0.61	5.04	<0.0001
ADHD-TD	–0.98	0.59	–1.67	0.2164
DLD-TD	–4.07	0.63	–6.42	<0.0001

**FIGURE 6:**
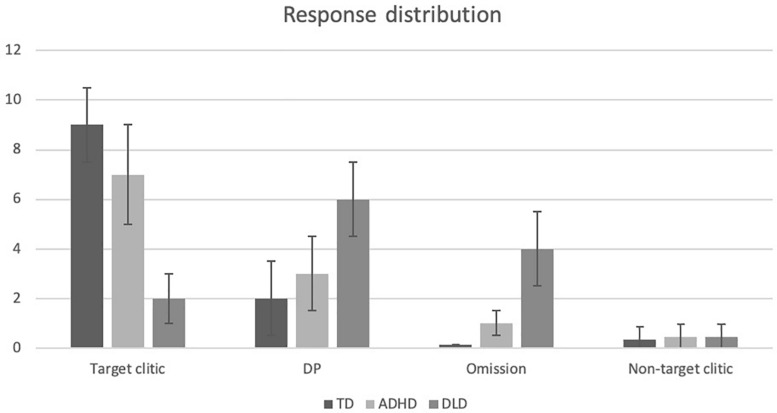
Response distribution for the probe test.

We also examined the other types of responses produced by our participants based on the coding criteria outlined in 2.1, once again using logistic regression analysis in a mixed model with cognitive groups as fixed factors and items and subjects as random factors. These analyses showed a main effect of cognitive group for the production of DPs, χ^2^(2,*N* = 60) = 16.67, *p* ≤ 0.001, with children with DLD producing significantly more DPs than TD children and children with ADHD. A significant main effect of cognitive group was also observed for the omission of clitics, χ^2^(2, *N* = 60) = 30.54, *p* ≤ 0.001, with TD children and children with ADHD performing similarly to one another but differently to children with DLD, the latter providing significantly more ungrammatical responses in which an object clitic had been omitted. In what concerns the production of non-target object clitics, no significant effect of cognitive group emerged, χ^2^(2, *N* = 60) = 0.57, *p* ≥ 0.05. This is likely due to the fact that very few errors of this type were made by the participants. These findings are summarized in Figure 6 and Table 6.

**TABLE 6:** Summary of *post hoc* Tukey test results for DP production and omitted object clitics following the mixed models for logistic regression analyses.

Contrast	Estimate	*SE*	*z*-ratio	*p*-value
**DP**				
ADHD-DLD	–1.30	0.47	–2.79	0.0147
ADHD-TD	0.61	0.49	1.24	0.4282
DLD-TD	1.91	0.48	3.95	0.0002
**Omission**				
ADHD-DLD	–2.13	0.56	–3.81	0.0004
ADHD-TD	1.54	0.75	2.07	0.0971
DLD-TD	3.67	0.72	5.08	<0.0001

### Question 2: Do Children With ADHD Perform Similarly on Both Morphosyntax Tasks?

Finally, as there was such a large degree of variability in the morphosyntactic performance of children with ADHD, we examined whether children in this group who performed poorly on the standardized omnibus test also performed poorly on the omnibus test. After controlling for age and non-verbal reasoning, partial correlations revealed a significant positive correlation between these two tasks for the ADHD group, *r*(17) = 0.64, *p* = 0.004. As previously mentioned, nine of the 20 children with ADHD scored at least 1.5 SDs below age norms on the omnibus test. The probe task we used is not a norm-referenced test, but we classified poor performance as a number of correct target clitics that was ≤5 based on the fact that the TD average was 9 with a SD of 3. Using this index, eight children with ADHD performed poorly on the probe task, five of whom also performed poorly on the probe test. These results are summarized in Table 7 and our interpretations of these results are discussed below.

**TABLE 7:** Descriptive data on participants with ADHD and summary of their performance on the two morphosyntax tasks.

Participants	Age (y;m)	Gender	ADHD profile	Comorbid difficulties	Omnibus z-score	Correct target clitics
1	9;4	m	Mixed	Suspected DLD	**−3.13**	**2**
2	6;3	m	Mixed	na	–0.69	**5**
3	6;6	m	Mixed	na	0.74	**1**
4	10;6	f	Mixed	na	0.10	10
5	10;2	m	Mixed	na	**−2.81**	11
6	7;10	f	Mixed	na	2.10	12
7	10;0	m	Mixed	Dyslexia	–1.05	10
8	7;8	f	Mixed	na	**−2.40**	9
9	9;6	m	Mixed	na	0.74	12
10	7;1	f	Mixed	Suspected DLD	**−1.90**	**3**
11	9;6	m	Mixed	Dyslexia	**−1.83**	6
12	10;7	m	Mixed	na	1.65	10
13	10;4	f	Mixed	Dyslexia	–0.55	12
14	9;2	m	Mixed	na	**−6.00**	**1**
15	8;0	m	Mixed	na	**−3.92**	**4**
16	8;9	f	Mixed	na	3.58	11
17	8;1	m	Hyperactive	na	**−3.50**	**5**
18	10;0	m	Mixed	na	**−1.86**	7
19	10;7	f	Mixed	na	0.84	12
20	7;3	m	Hyperactive	na	1.10	**4**

*Scores in bold indicate poor performance.*

## Discussion

Children with ADHD and DLD have both been associated with EF and morphosyntactic weakness and differentiating the two populations is sometimes a challenge for practitioners involved in their evaluation (Redmond, 2005, 2016; Redmond et al., 2011). The two aims of this study were (i) to compare the performance of TD children, children with ADHD and children with DLD on various EF and morphosyntax tasks to see if similar profiles emerge in the clinical groups, and (ii) to investigate how children with ADHD perform on two different types of morphosyntactic measures, an omnibus test that assessed the production of a variety of unpredictable morphosyntactic structures and a more fine-grained probe test that assessed the production of structures containing a 3p object clitic pronoun. These two tasks were chosen as standardized omnibus tests are frequently employed for diagnostic purposed by SLTs, and the probe task assessed a specific structure that is a clinical marker of DLD in French, although this type of task is not commonly used as a diagnostic tool.

Concerning the EF tasks, a distinct TD > ADHD > DLD pattern was observed for all six measures, with TD children consistently demonstrating the best performance of the three groups, children with DLD displaying the poorest performance, and children with ADHD performing between the TD and DLD groups. Indeed, TD children significantly outperformed children with DLD for five of the six measures, whereas this was the case for only two of six measures when TD-ADHD performance was compared. ADHD EF performance was linked to non-verbal reasoning for three of the six EF measures, but it is important to note that for the two EF measures with which the ADHD group demonstrated the most difficulty, performance was not moderated by non-verbal reasoning ability. Non-verbal reasoning was never linked to EF performance in the other two cognitive groups.

Looking specifically at the selective attention data, the performance of all three groups was close to ceiling. The fact that children with ADHD did not differ from TD children on this task is not necessarily surprising as studies assessing selective attention in ADHD have produced mixed results. Furthermore, in a study examining the utility of the TEA-ch battery from which we took our selective attention measure, Heaton et al. (2001) reported that the performance of English-speaking children with ADHD (similar in age to our own participants) did not differ from that of the control group on the selective attention task.

As for children with DLD, Ebert and Kohnert’s (2011) meta-analysis and Kapa and Plante’s (2015) literature review of EF capacity in children with DLD suggest that selective attention deficits are characteristic of this population. However, the selective attention measures considered in these reviews combined selective and sustained attention, and it is therefore possible that children with DLD in these studies struggled with the continuous aspect of the tasks rather than with the aspect of directing attention toward relevant stimuli while ignoring distracting information, which is what our task required them to do. Children with DLD did, however, significantly differ from TD children for selective attention speed, with the DLD group taking almost twice as long on average to find each target than TD children (10.16 s per target for children with DLD as opposed to 5.76 s per target for TD children). This could reflect coordination difficulties, but the TD-DLD difference remained even when the scores were adjusted for motor control. This is in line with other studies showing that children with DLD take longer to respond than TD children in both verbal and visuospatial tasks (Tallal and Piercy, 1973; Miller et al., 2001; Schul et al., 2004), which could reflect slower processing in children with DLD (Kail, 1994) or difficulties implementing an effective strategy during the visual search task (Anderson et al., 2001).

Only children with DLD demonstrated WM weakness, performing below both the TD and ADHD groups for simple span and below the TD group for complex span. These findings, which cannot be explained by poor non-verbal reasoning ability, are in line with a wealth of research reporting simple and complex span deficits in children with DLD. More interesting, perhaps, was the statistically significant ADHD-DLD difference for simple span. When Hutchinson et al. (2012) performed a similar comparison using the same simple and complex span tasks, they reported an identical pattern: only children with DLD performed poorly when assessed for simple span whereas both clinical groups performed poorly for complex span. That children with ADHD performed liked their TD peers on the simple span measure suggests that the storage/rehearsal mechanisms of WM are intact in this population. Their ability, however, to combine storage and processing, a higher-order EF skill, seems less stable, albeit not necessarily impaired, a finding also reported by Hutchinson et al. (2012). This is consistent with prior research showing that decreases in attention and higher activity levels in children with ADHD are more closely linked to the demands of complex WM tasks but less so to the demands of lower-level WM processes, such as simply maintaining information for later recall (Rapport et al., 2009; Kofler et al., 2010; Kasper et al., 2012). These findings suggest that children with ADHD and DLD can be distinguished based on performance on WM tests that tap into different WM skills, with WM deficits affecting both lower- and higher-order WM mechanisms in children with DLD, but only higher-order WM mechanisms in children with ADHD.

As for the attention shifting data, we found that both children with ADHD and DLD could be distinguished from their TD peers but that the two clinical groups could not be distinguished from one another. Previous work has reported variable findings for attention shifting capacity in children with ADHD, but our results corroborate earlier studies that have found attention shifting limitations in this population (Lawrence et al., 2004; Toplak et al., 2009; O’Brien et al., 2010). In particular, our results showed that children with ADHD not only struggled to perform our attention shifting task when they were asked to inhibit the conflicting prepotent responses triggered by the stimuli (the Opposite World condition), they also demonstrated significant difficulty in the Same World condition, which reflected the time it took the participants to process the mental task. As performance on the shifting task was not related to non-verbal reasoning, this suggests that attention shifting is impacted in ADHD regardless of IQ. The same conclusion can be made for children with DLD as our findings clearly indicate attention shifting weakness in children with DLD that is independent of non-verbal reasoning ability. Similarly to the ADHD group, children with DLD struggled with both the Same World and Opposite World conditions, which, again, seems to point to both processing and inhibition difficulties.

Concerning the morphosyntax tasks, a TD > ADHD > DLD pattern was observed for the standardized omnibus task, revealing three performance profiles that could all be statistically distinguished: a high-performing, unimpaired TD profile, a low-performing, impaired DLD profile, and an ADHD profile that demonstrated intermediate performance with a large degree of inter-individual variability that was related to non-verbal reasoning. The TD and DLD results are expected, and the results for children with ADHD are in line with previous research reporting morphosyntactic weakness in this population when standardized tests have been used to assess morphosyntax (Newcomer and Hammill, 1988; Cohen et al., 1993, 2000; Kim and Kaiser, 2000). Conversely, children with ADHD performed comparably to TD children on the probe test, with only the DLD group demonstrating impaired performance.

That children with ADHD performed comparably to their TD peers on the probe task that assessed the production of a clinical marker of French DLD seems to suggest that morphosyntactic impairment is not characteristic of ADHD. Factors related to test type, such as the necessity to switch back and forth between various complex structures, may penalize children with EF limitations (in particular in attention shifting), leading to low scores that mimic morphosyntactic weakness when omnibus tests that vary targets are used. Furthermore, poorer non-verbal reasoning skills in children with ADHD seem to contribute to poor performance on omnibus tests of morphosyntax. If morphosyntactic performance remains poor when using a test that optimally reduces external cognitive load factors, as was the case for the probe test, this likely indicates true morphosyntactic impairment. Therefore, children with ADHD in our study who performed poorly on both morphosyntax tasks may represent cases of comorbid ADHD and DLD, which is frequently reported in the literature (see Redmond, 2016 for an overview). However, it should be noted that results from different studies investigating comorbidity between ADHD and DLD are highly variable, with both high and low co-occurrence estimates being provided by researchers. It is therefore possible that such disparity in the literature is due precisely to the types of tests used to assess language, with low-specificity tests (e.g., omnibus tests) increasing the risk for diagnostic error and incorrectly identifying a large number of children with ADHD as having comorbid language deficits. The findings from this study should encourage SLTs to use both all-encompassing omnibus tests as well as probe tests that assess clinical markers of DLD when evaluating children with ADHD for morphosyntactic impairment. The former could be used as a measure of first detection, whereas the latter could serve diagnostic purposes.

Finally, this study reveals that WM capacity is potentially one key factor differentiating ADHD and DLD. Both children with ADHD and DLD performed poorly on the attention shifting measures but only children with DLD demonstrated WM vulnerability. There is robust empirical evidence demonstrating that WM deficits limit the ability of children with DLD to process complex morphosyntax (e.g., Delage and Frauenfelder, 2020), but it seems to be the case that deficits in attention shifting are insufficient to lead to the degree of morphosyntactic impairment associated with DLD. Otherwise, we would have expected children with ADHD to perform poorly on both morphosyntax tasks. A question that remains unanswered is whether or not it is the coupling of deficits in multiple EF components that engenders morphosyntactic impairment in DLD, or if weakness in WM is a sufficient condition for morphosyntax to be negatively impacted. Future studies should investigate this further.

## Study Limitations

The present study provides us with a better understanding of how the EF and morphosyntactic profiles of TD, ADHD and DLD children are similar and how they diverge, but this work also has several limitations. One limitation is the small sample sizes. The study should be replicated with a larger sample that would increase the reliability of the findings. The fact that nearly half of the DLD group was bilingual is another potential problem. While excluding bilinguals from participating in this type of research seems counterproductive, future work should aim to have a more balanced number of mono and bilingual participants in each of the groups tested. Obvious group differences on other potentially important variables, such as non-verbal reasoning, should also be avoided in future research. Finally, that a small number of children in our sample presented comorbid language and attention deficits is another limitation of this work. Replicating the study with children with comorbid difficulties as a separate group may generate more precise results.

## Data Availability Statement

The raw data supporting the conclusions of this article will be made available by the authors, without undue reservation.

## Ethics Statement

The studies involving human participants were reviewed and approved by (1) Commission cantonale de l’éthique de la recherche (Genève) (2) Commission d’éthique UniGe, Faculté de Psychologie et Sciences de l’Education. Written informed consent to participate in this study was provided by the participants’ legal guardian/next of kin.

## Author Contributions

ES conceived the present study, supervised the testing of the participants, prepared the data, ran the analyses, and drafted the manuscript. HD offered frequent supervisory support throughout the entire process and proofread various versions of the manuscript, providing abundant feedback and suggestions for improvement. All authors contributed to the final version of the manuscript.

## Conflict of Interest

The authors declare that the research was conducted in the absence of any commercial or financial relationships that could be construed as a potential conflict of interest.

## References

[B1] Alderson R. M. Rapport M. D. Hudec K. L. Sarver D. E. Kofler M. J. (2010). Competing core processes in attention-deficit/hyperactivity disorder (ADHD): do working memory deficiencies underlie behavioral inhibition deficits? *J. Abnorm. Child Psychol.* 38 497–507. 10.1007/s10802-010-9387-0 20140491

[B2] American Psychiatric Association (2013). *Diagnostic and Statistical Manual of Mental Disorders*, 5th Edn Washington, DC: American Psychiatric Publication.

[B3] Anderson V. A. Anderson P. Northam E. Jacobs R. Catroppa C. (2001). Development of executive functions through late childhood and adolescence in an Australian sample. *Dev. Neuropsychol.* 20 385–406. 10.1207/s15326942dn2001_5 11827095

[B4] Archibald L. M. Gathercole S. E. (2006). Short-term and working memory in specific language impairment. *Int. J. Lang. Commun. Disord.* 41 675–693. 10.1080/13682820500442602 17079222

[B5] Barkley R. A. (1997). Behavioral inhibition, sustained attention, and executive functions: constructing a unifying theory of ADHD. *Psychol. Bull.* 121 65–94. 10.1037/0033-2909.121.1.65 9000892

[B6] Biederman J. Petty C. R. Doyle A. E. Spencer T. Henderson C. S. Marion B. (2007). Stability of executive function deficits in girls with ADHD: a prospective longitudinal followup study into adolescence. *Devel. Neuropsychol.* 33 44–61. 10.1080/87565640701729755 18443969

[B7] Bishop D. (2003). *The Children’s Communication Checklist: CCC-2.* San Antonio, TX: Harcourt Assessment.

[B8] Bishop D. Snowling M. J. Thompson P. A. Greenhalgh T. Westerveld M. F. Murphy C-A. (2017). Phase 2 of CATALISE: a multinational and multidisciplinary Delphi consensus study of problems with language development: terminology. *J. Child Psychol. Psychiatry* 58 1068–1080. 10.1111/jcpp.12721 28369935 PMC5638113

[B9] Chillier-Zesiger L. Arabatzi M. Baranzini L. Cronel-Ohayon S. Thierry D. (2006). *The Acquisition of French Pronouns in Normal Children and in Children with Specific Language Impairment (SLI).* Geneva: University of Geneva.

[B10] Cohen N. J. Davine M. Horodezky N. Lipsett L. Isaacson L. (1993). Unsuspected language impairment in psychiatrically disturbed children: prevalence and language and behavioral characteristics. *J. Am. Acad. Child Adolesc. Psychiatry* 32 595–603. 10.1097/00004583-199305000-00016 8496124

[B11] Cohen N. J. Davine M. Meloche-Kelly M. (1989). Prevalence of unsuspected language disorders in a child psychiatric population. *J. Am. Acad. Child Adolesc. Psychiatry* 28 107–111. 10.1097/00004583-198901000-00020 2914822

[B12] Cohen N. J. Menna R. Vallance D. D. Barwick M. A. Im N. Horodezky N. (1998). Language, social cognitive processing, and behavioral characteristics of psychiatrically disturbed children with previously identified and unsuspected language impairments. *J. Child Psychol. Psychiatry* 39 853–864. 10.1017/s0021963098002789 9758194

[B13] Cohen N. J. Vallance D. D. Barwick M. Im N. Menna R. Horodezky N. (2000). The interface between ADHD and language impairment: an examination of language, achievement, and cognitive processing. *J. Child Psychol. Psychiatry* 41 353–362. 10784082

[B14] Conners C. K. (2008). *Conners Comprehensive Behavior Rating Scales (Conners CBRS).* Toronto, ON: Multi-Health Systems 10.1111/1469-7610.00619

[B15] Contemori C. Garraffa M. (2010). Comparison of modalities in SLI syntax: a study on the comprehension and production of non-canonical sentences. *Lingua* 120 1940–1955. 10.1016/j.lingua.2010.02.011

[B16] Cronel-Ohayon S. (2004). *Etude Longitudinale D’une Population D’enfants Francophones Présentant un Trouble Spécifique du Développement du Langage: Aspects Syntaxiques.* Doctoral dissertation, University of Geneva, Geneva.

[B17] Delage H. Durrleman S. Frauenfelder U. H. (2016). Disentangling sources of difficulty associated with the acquisition of accusative clitics in French. *Lingua* 180 1–24. 10.1016/j.lingua.2016.03.005

[B18] Delage H. Frauenfelder U. H. (2020). Relationship between working memory and complex syntax in children with developmental language disorder. *J. Child Lang.* 1–33. 10.1017/S0305000919000722 31775942

[B19] Delage H. Monjauze C. Hamann C. Tuller L. (2008). “Relative clauses in atypical acquisition of French,” in *Language acquisition and development: Proceedings of GALA 2007*, eds Gavarró A. Freitas J. (Newcastle: Cambridge Scholars Publishing), 166–176.

[B20] DeShazo Barry T. Klinger L. G. Lyman R. D. Bush D. Hawkins L. (2001). Visual selective attention versus sustained attention in boys with attention-deficit/hyperactivity disorder. *J. Atten. Disord.* 4 193–202. 10.1177/108705470100400401

[B21] Ebert K. D. Kohnert K. (2011). Sustained attention in children with primary language impairment: a meta-analysis. *J. Speech Lang. Hear. Res.* 54 1372–1384. 10.1044/1092-4388(2011/10-0231) 21646419 PMC4047633

[B22] Farrant B. M. Maybery M. T. Fletcher J. (2012). Language, cognitive flexibility, and explicit false belief understanding: longitudinal analysis in typical development and specific language impairment. *Child Dev.* 83 223–235. 10.1111/j.1467-8624.2011.01681.x 22188484

[B23] Fortea I. B. Forner C. B. Colomer C. Casas A. M. Miranda B. R. (2018). Communicative skills in Spanish children with autism spectrum disorder and children with attention deficit hyperactivity disorder. analysis through parents’ perceptions and narrative production. *Res. Autism Spectr. Disord.* 50 22–31. 10.1016/j.rasd.2018.02.006

[B24] Frizelle P. Fletcher P. (2014). Relative clause constructions in children with specific language impairment. *Int. J. Lang. Commun. Disord.* 49 255–264. 10.1111/1460-6984.12070 24304939

[B25] Geurts H. M. Embrechts M. (2008). Language profiles in ASD, SLI, and ADHD. *J. Autism Dev. Disord* 38 1931–1943. 10.1007/s10803-008-0587-1 18521730

[B26] Goldberg M. C. Mostofsky S. H. Cutting L. E. Mahone E. M. Astor B. C. Denckla M. B. (2005). Subtle executive impairment in children with autism and children with ADHD. *J. Autism Dev. Disord.* 35 279–293. 10.1007/s10803-005-3291-4 16119469

[B27] Hamann C. (2006). Speculations about early syntax: the production of wh-questions by normally developing French children and French children with SLI. *Catalan J. Linguist.* 5 143–189. 10.5565/rev/catjl.82

[B28] Heaton S. C. Reader S. K. Preston A. S. Fennell E. B. Puyana O. E. Gill N. (2001). The Test of Everyday Attention for Children (TEA-Ch): patterns of performance in children with ADHD and clinical controls. *Child Neuropsychol.* 7 251–264. 10.1076/chin.7.4.251.8736 16210214

[B29] Helland W. A. Biringer E. Helland T. Heimann M. (2012). Exploring language profiles for children with ADHD and children with Asperger syndrome. *J. Atten. Disord.* 16 34–43. 10.1177/1087054710378233 20837976

[B30] Henry L. A. Messer D. J. Nash G. (2012). Executive functioning in children with specific language impairment. *J. Child Psychol. Psychiatry* 53 37–45. 10.1111/j.1469-7610.2011.02430.x 21668446

[B31] Hutchinson E. Bavin E. Efron D. Sciberras E. (2012). A comparison of working memory profiles in school-aged children with specific language impairment, attention deficit/hyperactivity disorder, comorbid SLI and ADHD and their typically developing peers. *Child Neuropsychol.* 18 190–207. 10.1080/09297049.2011.601288 21919558

[B32] Im-Bolter N. Johnson J. Pascual-Leone J. (2006). Processing limitations in children with specific language impairment: the role of executive function. *Child Dev.* 77 1822–1841. 10.1111/j.1467-8624.2006.00976.x 17107463

[B33] Irwin L. N. Kofler M. J. Soto E. F. Groves N. B. (2019). Do children with attention-deficit/hyperactivity disorder (ADHD) have set shifting deficits? *Neuropsychology* 33 470–481. 10.1037/neu0000546 30945912 PMC6668027

[B34] Jacobson P. F. (2012). The effects of language impairment on the use of direct object pronouns and verb inflections in heritage Spanish speakers: a look at attrition, incomplete acquisition and maintenance. *Bilingualism: Lang. Cogn.* 15 22–38. 10.1017/S1366728911000484

[B35] Jakubowicz C. (2011). Measuring derivational complexity: new evidence from typically developing and SLI learners of L1 French. *Lingua* 121 339–351. 10.1016/j.lingua.2010.10.006

[B36] Jensen De López K. J. Olsen L. S. Chondrogianni V. (2014). Annoying Danish relatives: comprehension and production of relative clauses by Danish children with and without SLI. *J. Child Lang.* 41 51–83. 10.1017/s0305000912000517 23200200 PMC3866992

[B37] Kail R. (1994). A method for studying the generalized slowing hypothesis in children with specific language impairment. *J. Speech Lang. Hear. Res.* 37 418–421. 10.1044/jshr.3702.418 8028323

[B38] Kapa L. L. Plante E. (2015). Executive function in SLI: recent advances and future directions. *Curr. Dev. Disord. Rep.* 2 245–252. 10.1007/s40474-015-0050-x 26543795 PMC4629777

[B39] Kapa L. L. Plante E. Doubleday K. (2017). Applying an integrative framework of executive function to preschoolers with specific language impairment. *J. Speech Lang. Hear. Res.* 60 2170–2184. 10.1044/2017_jslhr-l-16-0027 28724132 PMC5829800

[B40] Kasper L. J. Alderson R. M. Hudec K. L. (2012). Moderators of working memory deficits in children with attention-deficit/hyperactivity disorder (ADHD): a meta-analytic review. *Clin. Psychol. Rev.* 32 605–617. 10.1016/j.cpr.2012.07.001 22917740

[B41] Khomsi A. Khomsi J. Pasquet F. Parbeau-Guéno A. (2007). *Bilan Informatisé de Langage Oral au Cycle 3 et au Collège (BILO-3C).* Paris: Éditions du Centre de psychologie appliquée.

[B42] Kim O. H. Kaiser A. P. (2000). Language characteristics of children with ADHD. *Commun. Disorde. Q.* 21 154–165. 10.1177/152574010002100304

[B43] Kofler M. J. Rapport M. D. Bolden J. Sarver D. E. Raiker J. S. (2010). ADHD and working memory: the impact of central executive deficits and exceeding storage/rehearsal capacity on observed inattentive behavior. *J. Abnorm. Child Psychol.* 38 149–161. 10.1007/s10802-009-9357-6 19787447

[B44] Korrel H. Mueller K. L. Silk T. Anderson V. Sciberras E. (2017). Research Review: language problems in children with Attention-Deficit Hyperactivity Disorder–a systematic meta-analytic review. *J. Child Psychol. Psychiatry* 58 640–654. 10.1111/jcpp.12688 28186338

[B45] Lawrence V. Houghton S. Douglas G. Durkin K. Whiting K. Tannock R. (2004). Executive function and ADHD: a comparison of children’s performance during neuropsychological testing and real-world activities. *J. Atten. Disord.* 7 137–149. 10.1177/108705470400700302 15260171

[B46] Leonard L. B. (2014). *Children with Specific Language Impairment.* Cambridge, MA: MIT press.

[B47] Love A. J. Thompson M. G. (1988). Language disorders and attention deficit disorders in young children referred for psychiatric services: analysis of prevalence and a conceptual synthesis. *Am. J Orthopsychiatry* 58 52–64. 10.1111/j.1939-0025.1988.tb01566.x 3257845

[B48] Majerus S. Leclercq A. L. Grossmann A. Billard C. Touzin M. van der Linden M. (2009). Serial order short-term memory capacities and specific language impairment: no evidence for a causal association. *Cortex* 45 708–720. 10.1016/j.cortex.2008.10.006 19084832

[B49] Manly T. Robertson I. H. Anderson V. Nimmo-Smith I. Lussier F. Flessas J. (2006). *TEA-Ch: Test d’Evaluation de l’Attention Chez L’enfant.* Paris: Éditions du Centre de psychologie appliquée (ECPA).

[B50] Martinussen R. Hayden J. Hogg-Johnson S. Tannock R. (2005). A meta-analysis of working memory impairments in children with attention-deficit/hyperactivity disorder. *J. Am. Acade. Child Adolesc. Psychiatry* 44 377–384. 10.1097/01.chi.0000153228.72591.73 15782085

[B51] Miller C. A. Kail R. Leonard L. B. Tomblin J. B. (2001). Speed of processing in children with specific language impairment. *J. Speech Lang. Hear. Res.* 44 416–433. 11324662 10.1044/1092-4388(2001/034)

[B52] Mulas F. Capilla A. Fernández S. Etchepareborda M. C. Campo P. Maestú F. (2006). Shifting-related brain magnetic activity in attention-deficit/hyperactivity disorder. *Biol. Psychiatry* 59 373–379. 16154541 10.1016/j.biopsych.2005.06.031

[B53] Newcomer P. Hammill D. (1988). *Test of Language Development—2 Primary.* Austin, TX: Pro-Ed.

[B54] Novogrodsky R. Friedmann N. (2006). The production of relative clauses in syntactic SLI: a window to the nature of the impairment. *Adv. Speech Lang. Pathol.* 8 364–375. 10.1080/14417040600919496

[B55] O’Brien J. W. Dowell L. R. Mostofsky S. H. Denckla M. B. Mahone E. M. (2010). Neuropsychological profile of executive function in girls with attention-deficit/hyperactivity disorder. *Arch. Clin. Neuropsychol.* 25 656–670. 10.1093/arclin/acq050 20639299 PMC2957961

[B56] Paradis J. Crago M. Genesee F. Beachley B. Brown A. Conlin F. (2003). “Object clitics as a clinical marker of SLI in French: evidence from french-english bilingual children,” in *Proceedings of the 27th Annual Boston University Conference on Language Development*, Vol. 2 (Somerville, MA: Cascadilla Press), 638–649.

[B57] Purvis K. L. Tannock R. (1997). Language abilities in children with attention deficit hyperactivity disorder, reading disabilities, and normal controls. *J. Abnorm. Child Psychol.* 25 133–144. 9109030 10.1023/a:1025731529006

[B58] R Core Team (2020). *R: A Language and Environment for Statistical Computing.* R Foundation for Statistical Computing.

[B59] Rapport M. D. Bolden J. Kofler M. J. Sarver D. E. Raiker J. S. Alderson R. M. (2009). Hyperactivity in boys with attention-deficit/hyperactivity disorder (ADHD): a ubiquitous core symptom or manifestation of working memory deficits? *J. Abnorm. Child Psychol.* 37 521–534. 10.1007/s10802-008-9287-8 19083090

[B60] Raven J. Raven J. C. Court J. H. (1998). *Manual for Raven’s Progressive Matrices and Vocabulary Scales.* Oxford: Oxford Psychologists Press.

[B61] Redmond S. M. (2005). Differentiating SLI from ADHD using children’s sentence recall and production of past tense morphology. *Clin. Linguist. Phon.* 19 109–127. 10.1080/02699200410001669870 15704501

[B62] Redmond S. M. (2016). Markers, models, and measurement error: exploring the links between attention deficits and language impairments. *J. Speech Lang. Hear. Res.* 59 62–71. 10.1044/2015_jslhr-l-15-0088 26501406 PMC4867933

[B63] Redmond S. M. Thompson H. L. Goldstein S. (2011). Psycholinguistic profiling differentiates specific language impairment from typical development and from attention-deficit/hyperactivity disorder. *J. Speech Lang. Hear. Res.* 54 99–117. 10.1044/1092-4388(2010/10-0010) 20719871 PMC4493886

[B64] Reynell J. Curwen M. P. (1977). *Manual for the Reynell Developmental Language Scales (revised).* Slough: NFER.

[B65] Richards G. P. Samuels S. J. Turnure J. E. Ysseldyke J. E. (1990). Sustained and selective attention in children with learning disabilities. *J. Learn. Disabil.* 23 129–136. 10.1177/002221949002300210 2303740

[B66] Roello M. Ferretti M. L. Colonnello V. Levi G. (2015). When words lead to solutions: executive function deficits in preschool children with specific language impairment. *Res. Dev. Disabil.* 37 216–222. 10.1016/j.ridd.2014.11.017 25528081

[B67] Schul R. Stiles J. Wulfeck B. Townsend J. (2004). How ‘generalized’is the ‘slowed processing’in SLI? The case of visuospatial attentional orienting. *Neuropsychologia* 42 661–671. 10.1016/j.neuropsychologia.2003.10.010 14725803

[B68] Semel E. M. Wiig E. H. Secord W. (1995). *CELF3: Clinical Evaluation of Language Fundamentals.* San Diego, CA: Harcourt Brace.

[B69] Shalev L. Tsal Y. (2003). The wide attentional window: a major deficit of children with attention difficulties. *J. Learn. Disabil.* 36 517–527. 10.1177/00222194030360060301 15493434

[B70] Stanford E. Durrleman S. Delage H. (2019). The effect of working memory training on a clinical marker of French-speaking children with developmental language disorder. *Am. J. Speech Lang. Pathol.* 28 1388–1410. 10.1044/2019_ajslp-18-0238 31419156

[B71] Stavrakaki S. (2001). Comprehension of reversible relative clauses in specifically language impaired and normally developing Greek children. *Brain Lang.* 77 419–431. 10.1006/brln.2000.2412 11386707

[B72] Tallal P. Piercy M. (1973). Defects of non-verbal auditory perception in children with developmental aphasia. *Nature* 241 468–469. 10.1038/241468a0 4705758

[B73] Tannock R. M. Schachar R. (1996). “Executive dysfunction as an underlying mechanism of behavior and language problems in attention deficit hyperactivity disorder,” in *Language, Learning, and Behavior Disorders: Developmental, Biological, and Clinical Perspectives*, eds Beitchman J. H. Cohen N. J. Konstantareas M. M. Tannock R. (Cambridge: Cambridge University Press), 128–155.

[B74] Tirosh E. Cohen A. (1998). Language deficit with attention-deficit disorder: a prevalent comorbidity. *J. Child Neurol.* 13 493–497. 10.1177/088307389801301005 9796755

[B75] Toplak M. E. Bucciarelli S. M. Jain U. Tannock R. (2008). Executive functions: performance-based measures and the behavior rating inventory of executive function (BRIEF) in adolescents with attention deficit/hyperactivity disorder (ADHD). *Child Neuropsychol.* 15 53–72. 10.1080/09297040802070929 18608232

[B76] Toplak M. E. Pitch A. Flora D. B. Iwenofu L. Ghelani K. Jain U. (2009). The unity and diversity of inattention and hyperactivity/impulsivity in ADHD: evidence for a general factor with separable dimensions. *J. Abnorm. Psychol.* 37 1137–1150. 10.1007/s10802-009-9336-y 19562477

[B77] Tuller L. Delage H. Monjauze C. Piller A. G. Barthez M. A. (2011). Clitic pronoun production as a measure of atypical language development in French. *Lingua* 121 423–441. 10.1016/j.lingua.2010.10.008

[B78] Väisänen R. Loukusa S. Moilanen I. Yliherva A. (2014). Language and pragmatic profile in children with ADHD measured by children’s communication checklist 2nd edition. *Logoped. Phoniatr. Vocol.* 39 179–187. 24580020 10.3109/14015439.2013.784802

[B79] Wechsler D. (2003). *Wechsler Intelligence Scale for Children.* San Antonio, TX: The psychological corporation.

[B80] Weismer S. E. Evans J. Hesketh L. J. (1999). An examination of verbal working memory capacity in children with specific language impairment. *J. Speech Lang. Hear. Res.* 42 1249–1260. 10.1044/jslhr.4205.1249 10515519

[B81] Willcutt E. G. Doyle A. E. Nigg J. T. Faraone S. V. Pennington B. F. (2005). Validity of the executive function theory of attention-deficit/hyperactivity disorder: a meta-analytic review. *Biol. Psychiatry* 57 1336–1346. 10.1016/j.biopsych.2005.02.006 15950006

